# Morphological and morphometric characteristics of the nasopalatine canal in dentate and completely edentulous patients: a cone-beam computed tomography cross-sectional study

**DOI:** 10.3389/froh.2026.1899731

**Published:** 2026-07-22

**Authors:** Cam Le Ngoc Hong, Nhung Hong Nguyen, Quynh Thi Nhu Ngo, Tram Nguyen Phuong Pham, Ngoc Thi Thanh Nguyen, Tu Lam Doan

**Affiliations:** Faculty of Odonto-Stomatology, University of Health Sciences, Vietnam National University, Ho Chi Minh City, Vietnam

**Keywords:** cone-beam computed tomography (CBCT), dental implants (MeSH), edentulous maxilla, incisive canal, nasopalatine canal (NPC)

## Abstract

**Background:**

Anatomical changes associated with tooth loss may alter the morphology and dimensions of the nasopalatine canal, potentially affecting implant planning. However, comparative evidence between dentate and completely edentulous maxillae remains limited. This study aimed to evaluate and compare the morphological and morphometric characteristics of the nasopalatine canal and the anterior bone region in dentate and completely edentulous patients via cone-beam computed tomography (CBCT).

**Methods:**

Cross-sectional CBCT analysis was performed on 108 patients (54 dentate maxillae and 54 completely edentulous maxillae). Morphometric parameters of the nasopalatine canal and the bone anterior to the canal were measured in the axial, sagittal, and coronal planes. Interobserver reliability was assessed via intraclass correlation coefficients (ICCs) and Cohen's kappa. Intergroup comparisons were conducted via regression models adjusted for age and sex.

**Results:**

Compared with the completely edentulous group, the dentate group presented a significantly greater nasopalatine canal length (mean difference: 1.66 mm; 95% CI: 0.42–2.90; *p* = 0.009), whereas canal angulation was smaller (−6.47°; 95% CI: −9.93 to −3.00; *p* < 0.001). On sagittal images, the middle and inferior canal widths were significantly smaller in the dentate group (ratios of means: 0.79 and 0.80; *p* < 0.001), whereas the superior width was not different. Similarly, on axial views, the middle and inferior horizontal canal widths were reduced in the dentate group. The bone region anterior to the nasopalatine canal in the dentate individuals presented greater buccal crestal length (4.66 mm; *p* < 0.001) and greater bone width at 1 mm and 6 mm apical to the crest (ratios: 1.65 and 1.24; *p* < 0.001). Morphologically, the Y-shaped canal configuration predominated in dentate patients (51.9%), whereas the single-canal configuration was more common in the completely edentulous group (64.8%). On the sagittal plane, cylindrical morphology was significantly more common in dentate individuals [odds ratio (OR) = 3.68; 95% CI: 1.56–9.00].

**Conclusions:**

Completely edentulous patients exhibit wider and more angulated canals but reduced canal length and anterior bone volume.

## 1 Background

Dental implant therapy has become a well-established and highly reliable method for replacing missing teeth, especially in areas with strict functional and aesthetic requirements, such as the anterior maxilla (1, 2). However, the anterior maxilla presents significant anatomical challenges. It is often affected by progressive alveolar bone loss following tooth extraction and contains several critical neurovascular structures. One important anatomical feature to consider during preoperative implant planning is the nasopalatine canal, which must be carefully assessed (3).

The nasopalatine canal is a midline anatomical structure of the maxilla that contains the nasopalatine nerve and accompanying vascular branches responsible for sensory innervation of the anterior palate. Clinically, the morphology, dimensions, trajectory, and spatial relationship of the nasopalatine canal with the surrounding anterior maxillary bone exhibit substantial interindividual variability. Such anatomical variations may directly influence the position and angulation of implant placement and may increase the risk of complications, including sensory disturbances, intraoperative hemorrhage, impaired osseointegration, or the development of nasopalatine duct cysts (4). Moreover, in cases of severe alveolar ridge resorption following complete edentulism of the maxilla, the nasopalatine canal and its surrounding structures have increasingly been utilized as anchorage sites for implant placement (5, 6).

Comprehensive assessment of the nasopalatine canal via cone-beam computed tomography (CBCT) is therefore essential for accurate planning of implant treatment, enabling clinicians to determine the most appropriate therapeutic approach while minimizing the risk of complications associated with implant placement in this region (7). Numerous CBCT-based studies have investigated the anatomical and morphometric characteristics of the nasopalatine canal because CBCT provides highly accurate three-dimensional visualization and quantitative assessment of anatomical structures (8–10). However, the majority of these studies have focused primarily on canal morphology in dentate populations (8, 9) or in edentulous patients alone (11). Other investigations have compared partially edentulous individuals with dentate subjects (8, 12, 13). In contrast, research specifically examining the nasopalatine canal in completely edentulous patients remains relatively scarce, and the few available studies are generally based on limited sample sizes (13).

Despite the increasing clinical importance of this anatomical region, the available literature remains limited regarding comprehensive evaluations of the nasopalatine canal in completely edentulous patients, particularly studies that directly compare these characteristics with those observed in dentate individuals. Therefore, the present study aimed to investigate and compare the anatomical features of the nasopalatine canal via CBCT in two distinct patient populations: individuals with a completely edentulous maxilla and those with preserved dentition.

## 2 Materials and methods

### 2.1 Study design and population

A descriptive cross-sectional study was performed using CBCT datasets obtained from 108 patients who underwent imaging at a private hospital between April 2024 and December 2025. The CBCT records were categorized into two groups according to dental status. The completely edentulous group consisted of 54 CBCT scans from patients with complete maxillary edentulism, whereas the dentate group included 54 CBCT scans from patients retaining natural dentition in the anterior maxillary region. Maxillae demonstrating the presence of natural teeth or residual root fragments within the anterior maxillary region were classified into the dentate group. Conversely, maxillae presenting with complete edentulism, or those retaining only a limited number of teeth but lacking anterior maxillary teeth, were assigned to the completely edentulous group.

### 2.2 Ethics approval

Ethical approval for this study was obtained from the Institutional Ethics Committee in Biomedical Research at the University of Health Sciences, Vietnam National University, Ho Chi Minh City, under approval number 02/QD-HDDD.

### 2.3 Sample selection

**Figure 1:**
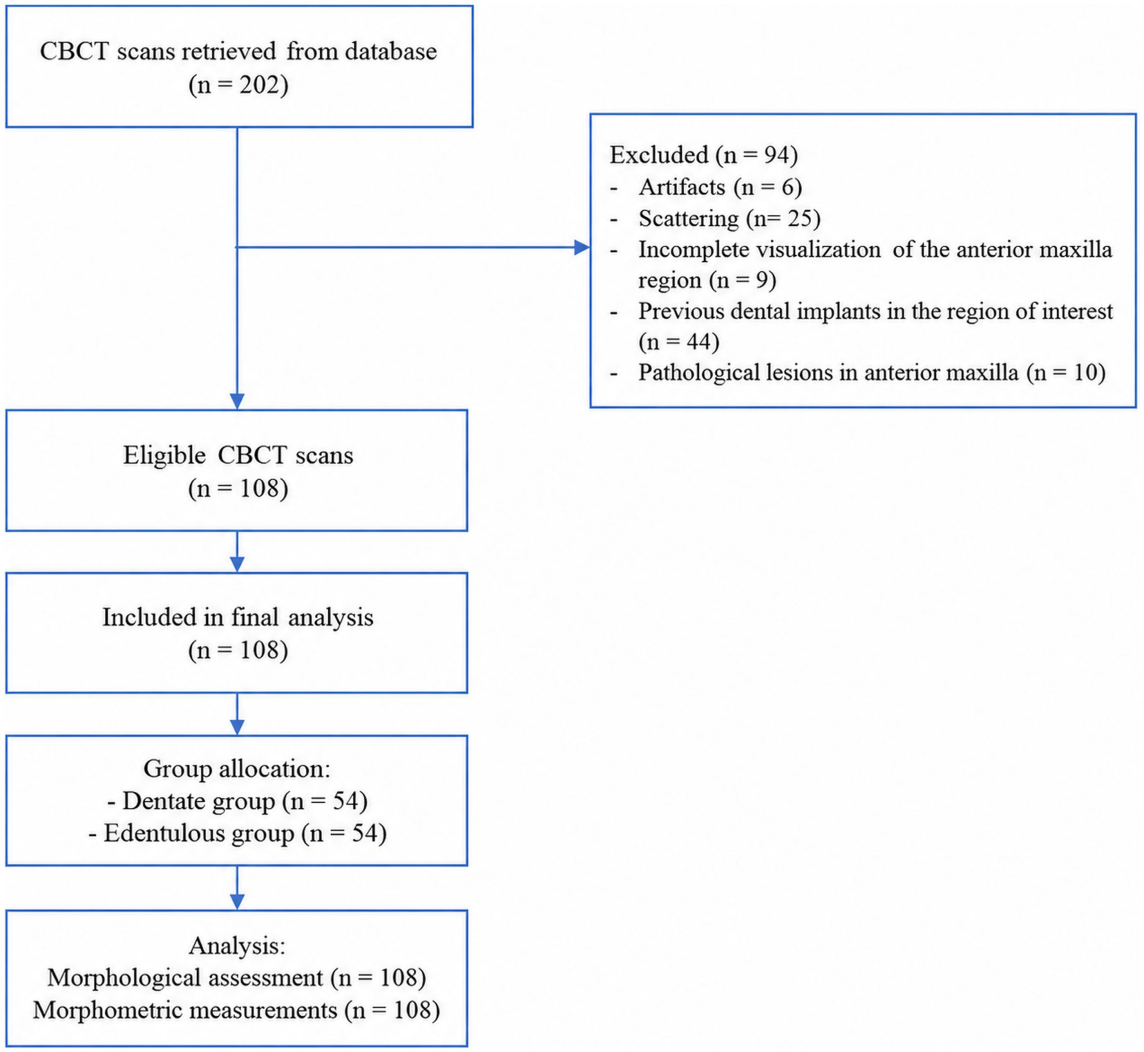
Flow diagram of CBCT scan selection and inclusion. Of 202 retrieved scans, 94 were excluded based on predefined criteria, resulting in 108 eligible scans included in the final analysis. The sample was equally divided into dentate (*n* = 54) and completely edentulous (*n* = 54) groups, with all cases subjected to morphological and morphometric assessment.

CBCT scans, including cases affected by artifacts, scattering, or incomplete visualization of the region of interest, were excluded if the image quality was insufficient for accurate evaluation. Images showing previously placed dental implants in the nasopalatine canal region or in the maxillary central incisor area were also excluded. In addition, scans demonstrating prior bone grafting procedures in the anterior maxilla were not considered. Patients presenting with radiographic evidence of pathological conditions, such as tumors or cystic lesions involving the nasopalatine canal or the maxillary incisor region, were excluded. Furthermore, CBCT images indicating bone defects in the nasopalatine canal region attributable to pathology, trauma, or previous surgical interventions within the area of interest were not included in the study. CBCT scans were retrieved from the institutional database. After applying predefined inclusion and exclusion criteria, eligible scans were selected for analysis (Figure 1).

### 2.4 Sample size calculation

Sample size estimation was performed to detect a clinically meaningful difference in the morphometric parameters of the nasopalatine canal between the dentate and completely edentulous groups. The calculation was based on data from a previous study (7) reporting mean values of 2.73 mm and 2.77 mm with standard deviations of 1.37 mm and 1.40 mm, respectively. The pooled standard deviation was therefore estimated at 1.385 mm. Assuming a minimum detectable difference of 0.75 mm, a two-sided significance level of 0.05, and a statistical power of 80%, the required sample size was calculated via the following formula for comparing two independent means:

$$n=\frac{2{\left(Z_{\alpha /2}+Z_{\beta }\right)}^{2}SD_{p}^{2}}{\delta^{2}}$$

*Z_α_*_/2_ = 1.96; *Z_β_* = 0.84, SD*_p_* = 1.385 and *δ* = 0.75. On the basis of these parameters, the required sample size was 54 participants per group (108 total).

The corresponding standardized effect size (Cohen's *d*) was 0.54, indicating a moderate effect. This sample size provides adequate statistical power to detect clinically relevant differences in the morphometric characteristics of the nasopalatine canal between the two study groups.

### 2.5 Radiographic imaging acquisition

CBCT examinations were performed via a dedicated cone-beam computed tomography unit (NewTom GiANO HR; Cefla, Imola, Italy). All scans were acquired via standardized imaging parameters in accordance with the manufacturer's recommendations. The acquisition protocol included a voxel size of 0.16 mm, a tube voltage of 90 kVp, and a tube current of 15 mA. The exposure time was set at 16.8 s, and a field of view (FOV) of 10 cm × 10 cm was selected to ensure complete visualization of the anterior maxillary region, including the nasopalatine canal and surrounding anatomical structures.

During CBCT scanning, patients were positioned in an upright posture with the chin resting on the chin support and the head stabilized to minimize motion artifacts. The radiology technician ensured proper alignment by orienting the patient according to the Frankfort horizontal plane and adjusting the reference beams to pass through the canine region. After image acquisition, the datasets were reviewed to confirm image quality and completeness. In cases where image acquisition errors or data storage failure occurred, the scan was repeated. All the CBCT datasets were exported and archived in Digital Imaging and Communications in Medicine (DICOM) format for subsequent analysis.

### 2.6 Image orientation and standardization

Prior to measurement, all the CBCT datasets were standardized to ensure consistent anatomical orientation. The images were reoriented using the Frankfort horizontal plane as the reference plane. The sagittal plane was adjusted to pass through the anterior nasal spine, whereas the coronal plane was aligned perpendicularly to the sagittal plane. This standardized orientation minimized measurement bias and ensured consistency across all evaluated scans.

Morphometric measurements and anatomical assessments of the nasopalatine canal and the bone region anterior to the canal were performed on axial, sagittal (sections passing through the anterior nasal spine), and coronal planes. All morphometric measurements were subsequently performed via the built-in digital measurement tools of the analysis software (Blue Sky Bio version 4.13, Blue Sky Bio, Libertyville, IL, USA), and values were recorded in millimeters (mm).

### 2.7 CBCT morphometric measurements

#### 2.7.1 Morphological and morphometric characteristics of the nasopalatine canal

**Figure 2:**
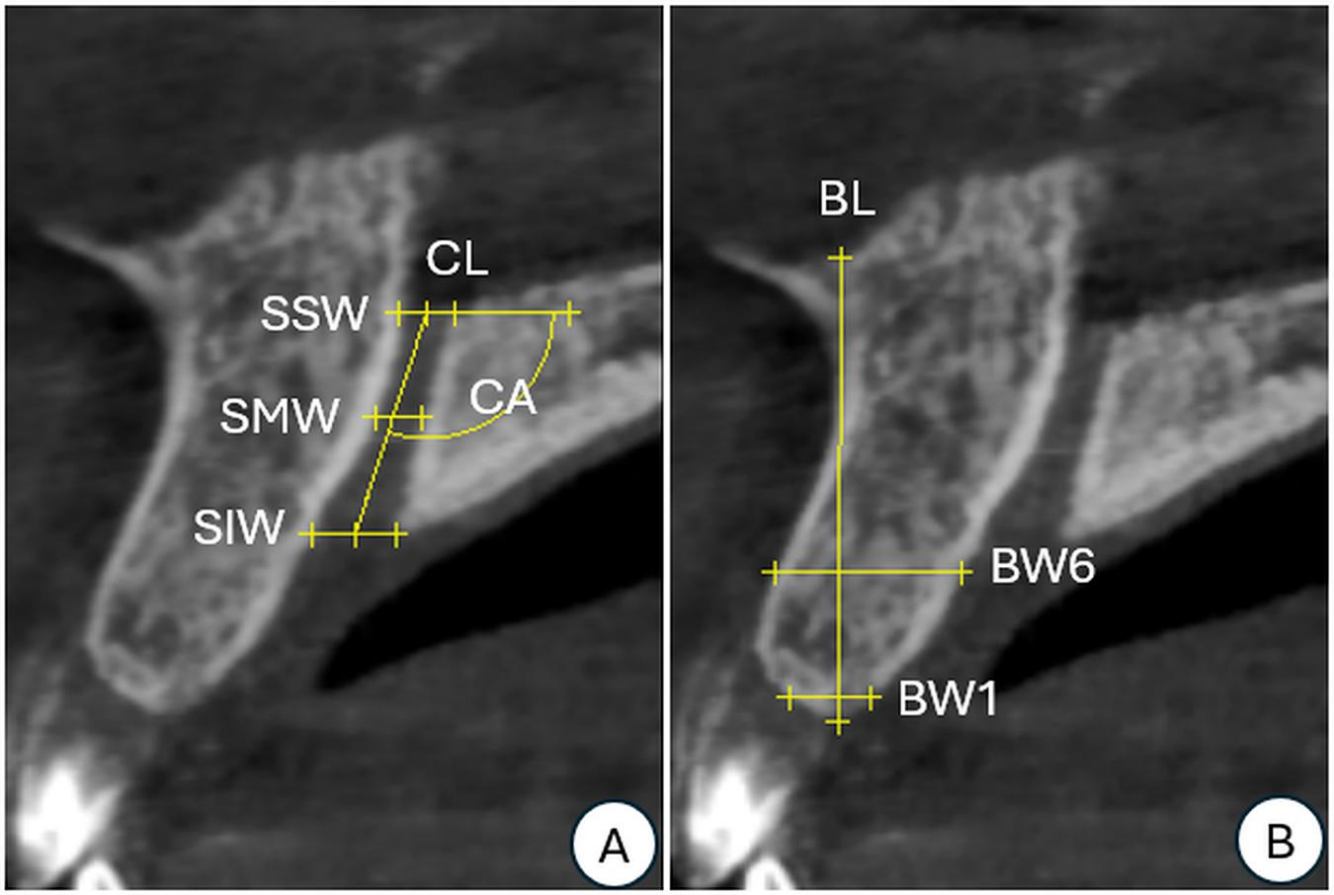
Measurements were obtained from sagittal sections passing through the anterior nasal spine. **(A)** Morphometric characteristics of the nasopalatine canal. **(B)** Morphometric characteristics of the bone anterior to the nasopalatine canal. SSW, sagittal superior canal width; SMW, sagittal middle canal width; SIW, sagittal inferior canal width; CL, canal length; CA, canal angulation; BW1, buccal bone width measured 1 mm apical to the alveolar crest; BW6, buccal bone width measured 6 mm apical to the alveolar crest; BL, buccal bone height.

**Figure 3:**
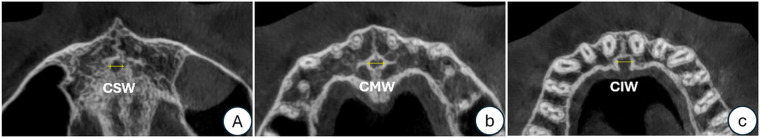
Measurements of horizontal canal width at different axial levels. CSW, horizontal superior canal width; CMW, horizontal middle canal width; CIW, horizontal inferior canal width.

**Figure 4:**
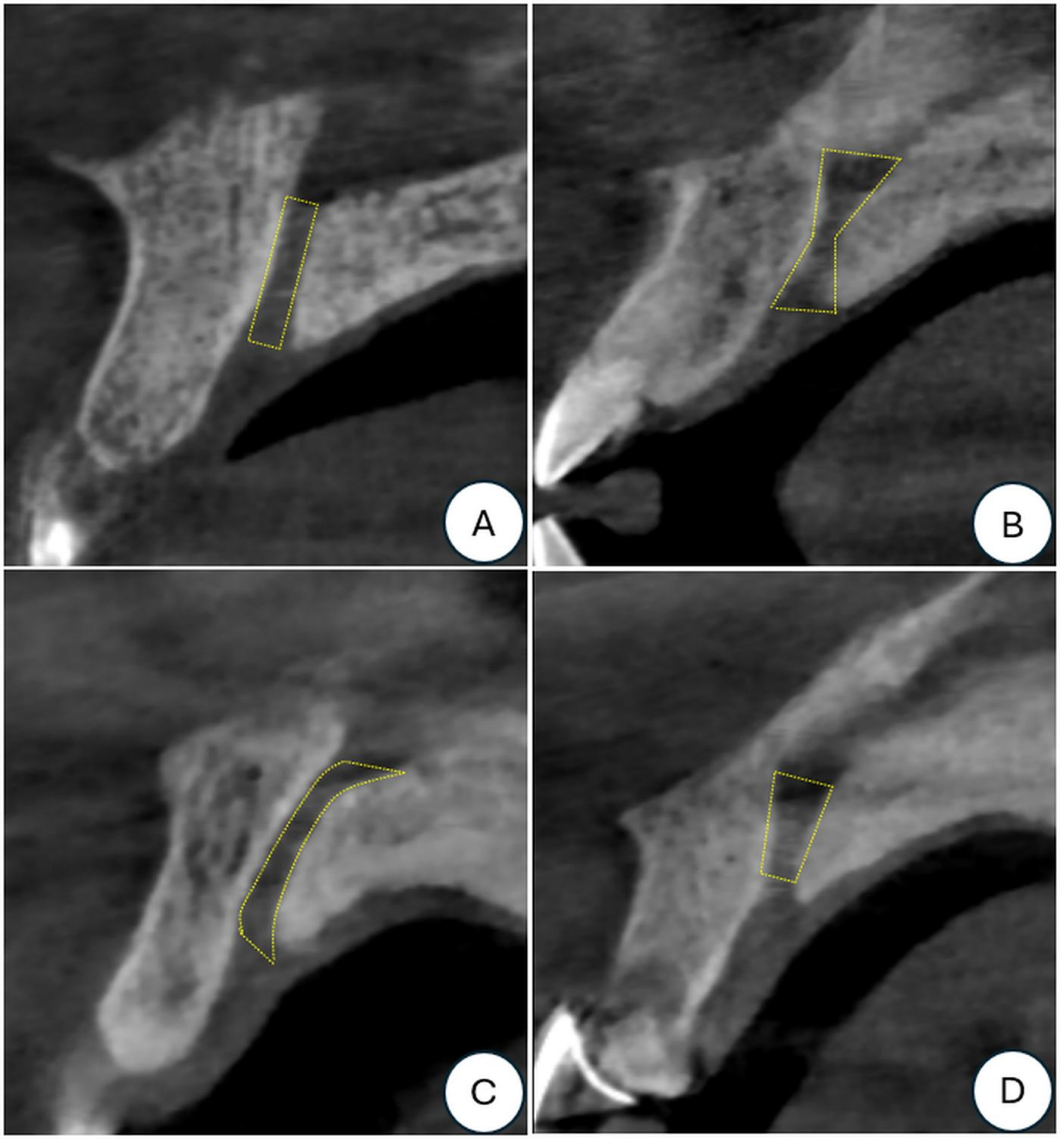
Morphological configurations of the nasopalatine canal in the sagittal plane: **(A)** cylindrical; **(B)** hourglass-shaped; **(C)** banana-shaped; **(D)** funnel-shaped.

**Figure 5:**
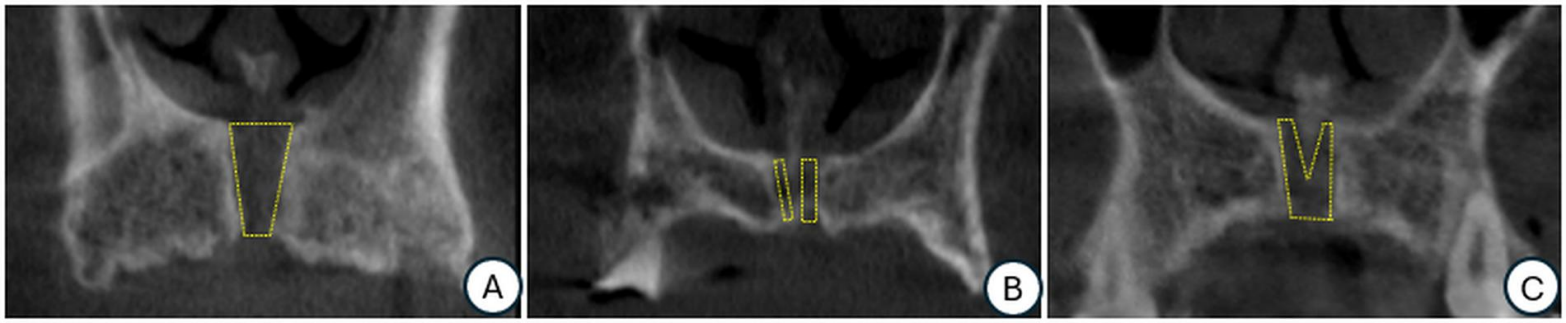
Morphological configurations of the nasopalatine canal in the coronal plane: **(A)** single and broad canal; **(B)** two parallel canals; **(C)** Y-shaped canal.

On the sagittal section passing through the anterior nasal spine (ANS), the sagittal canal width was measured at three levels—inferior, middle, and superior—parallel to the reference plane. The canal width measured at the nasal opening and palatal opening corresponded to the sagittal canal superior width and the sagittal canal inferior width, respectively. On the same sagittal section, the canal length was defined as the linear distance extending from the superior plane to the inferior plane along the longitudinal axis of the canal. The nasopalatine canal angle was determined as the angle formed between the canal axis and the reference plane. Figure 2A provides a schematic representation of the measurement procedures for the evaluated variables. The horizontal canal width was defined as the maximum canal width measured on axial sections at the superior, middle, and inferior levels (Figure 3). The morphology of the nasopalatine canal was classified according to its appearance on the sagittal plane into four categories: cylindrical, funnel-shaped, hourglass-shaped, and banana-shaped (Figure 4). On the coronal plane, canal morphology was categorized into three configurations: single and broad, two parallel canals, and Y-shaped canals (Figure 5).

#### 2.7.2 Buccal bone region of the nasopalatine canal

For the same sagittal section passing through the ANS, the buccal bone height was measured as the vertical distance from the alveolar crest to the nasal floor, perpendicular to the reference plane. The width of the buccal bone was measured at 1 mm and 6 mm apical to the alveolar crest toward the nasal floor (Figure 2B).

### 2.8 Observer calibration and measurement reliability

All morphometric measurements were independently performed by two calibrated examiners with experience in CBCT image analysis. Prior to the formal evaluation, both examiners underwent a calibration session to standardize the identification of anatomical landmarks and the measurement protocol within the analysis software. To assess intra- and interobserver reliability, 30 CBCT scans were randomly selected from the total dataset, including 15 scans from the dentate group and 15 scans from the completely edentulous group. These cases were re-evaluated independently by both examiners following the same measurement protocol. To assess the measurement reliability of the quantitative variables, the ICC was calculated via representative parameters, including the sagittal canal middle width, horizontal canal middle width, anterior bone height, and buccal bone width at 6 mm apical to the alveolar crest. For the qualitative variable of sagittal canal shape, interobserver agreement was evaluated via Cohen's kappa coefficient. The reliability coefficients were interpreted according to commonly accepted thresholds, where higher ICC and kappa values indicate stronger agreement between measurements.

### 2.9 Statistical analysis

All the statistical analyses were performed via R statistical software (R Foundation for Statistical Computing, Vienna, Austria). Continuous variables were assessed for normality via the Shapiro–Wilk test and graphical inspection (histograms and *Q*–*Q* plots). Intergroup comparisons of morphometric parameters of the nasopalatine canal between the dentate and edentulous groups were conducted while adjusting for age and sex. Variables with approximately normal distributions were analyzed via analysis of covariance (ANCOVA) with linear models to estimate adjusted mean differences. Positively skewed variables were analyzed via generalized linear models (GLMs) with a gamma distribution and log link, and the results are reported as ratios of adjusted means. The magnitude of the intergroup differences was quantified via standardized effect sizes (Cohen's *d* with Hedges' correction) and partial eta squared (partial *η*^2^). The morphological characteristics of the nasopalatine canal on the coronal and sagittal planes were summarized as frequencies and percentages. Differences in distribution between groups were assessed via the chi-square test or Fisher's exact test, followed by *post hoc* logistic regression to estimate ORs with 95% CIs for each configuration. Bonferroni correction was applied to account for multiple comparisons. *Post hoc* power analyses were performed for all morphometric outcomes using the observed effect sizes. A two-sided *p* value <0.05 was considered statistically significant.

## 3 Results

### 3.1 ICC and kappa

The ICC values ranged from 0.791 to 0.905, demonstrating good to excellent interobserver reliability for all the CBCT measurements. Cohen's kappa analysis demonstrated substantial interobserver agreement for the assessment of nasopalatine canal morphology in both imaging planes (coronal plane: *κ* = 0.789; sagittal plane: *κ* = 0.775; both *p* < 0.001).

### 3.2 Participant characteristics

A total of 108 CBCT scans from 108 patients (48 females and 60 males) were included in the analysis. The mean age of the participants in the completely edentulous group was 62.72 ± 10.23 years, whereas those in the dentate group had a mean age of 59.22 ± 10.20 years.

### 3.3 Morphometric characteristics of the nasopalatine canal

The length of the nasopalatine canal was significantly greater in the dentate group than in the completely edentulous group, with an adjusted mean difference of 1.66 mm (95% CI: 0.42–2.90; *p* = 0.009). In contrast, the angulation of the nasopalatine canal was significantly smaller in the dentate group, with an adjusted mean difference of −6.47° (95% CI: −9.93 to −3.00; *p* < 0.001).

On the sagittal plane, no significant difference was observed in the superior sagittal canal width between the two groups (ratio of means = 0.95, 95% CI: 0.80–1.12; *p* = 0.522). However, the middle sagittal canal width and inferior sagittal canal width were significantly smaller in the dentate group than in the completely edentulous group, with adjusted ratios of 0.79 (95% CI: 0.69–0.91; *p* = 0.001) and 0.80 (95% CI: 0.73–0.87; *p* < 0.001), corresponding to approximately 21% and 20% lower values, respectively.

On the axial plane, the superior horizontal canal width did not differ significantly between the two groups (mean ratio = 1.10, 95% CI: 0.98–1.25; *p* = 0.122). In contrast, both the middle and the inferior horizontal canal widths were significantly smaller in the dentate group. The adjusted ratio of the means for the middle horizontal canal width was 0.85 (95% CI: 0.75–0.97; *p* = 0.017), whereas the inferior horizontal canal width showed an adjusted mean difference of −1.04 mm (95% CI: −1.46 to −0.63; *p* < 0.001).

**Figure 6:**
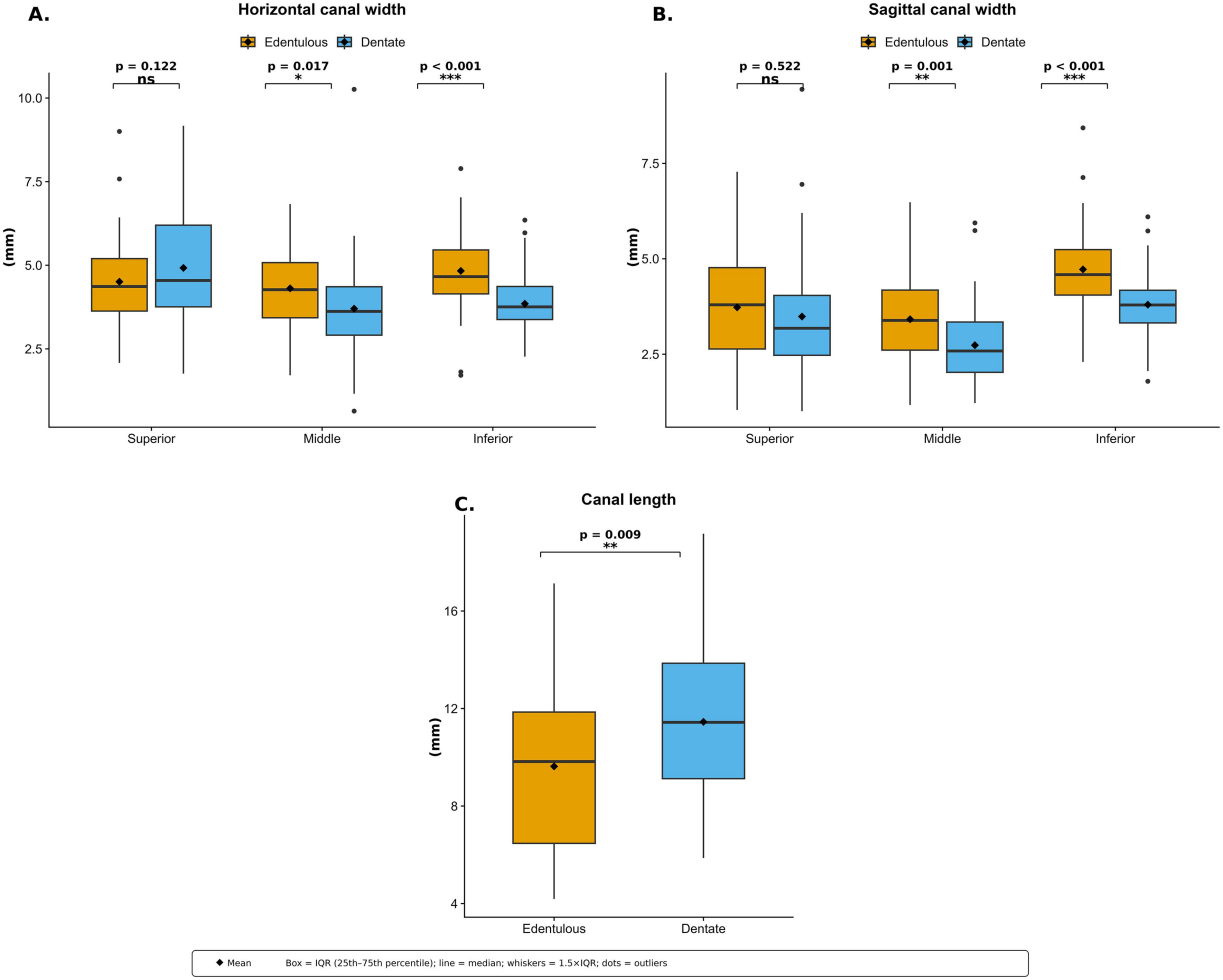
Comparison of the morphometric characteristics of the nasopalatine canal between dentate and completely edentulous participants. **(A)** Horizontal canal widths measured at the superior, middle, and inferior levels. **(B)** Sagittal canal widths measured at the superior, middle, and inferior levels. **(C)** Canal length. ns: not statistically significant; *, **, ***: statistically significant.

The detailed results of the morphometric characteristics of the nasopalatine canal are presented in Table 1 and Figure 6.

**Table 1:** Morphometric characteristics of the nasopalatine canal.

Variable	Completely edentulous group (mean ± SD) (mm)	Dentate group (mean ± SD) (mm)	Estimate	95% CI	*P* value	Model
Superior horizontal canal width	4.51 ± 1.34	4.92 ± 1.69	1.10	0.98 to 1.25	0.122	GLM
Middle horizontal canal width	4.31 ± 1.15	3.70 ± 1.45	0.85	0.75 to 0.97	0.017	GLM
Inferior horizontal canal width	4.83 ± 1.24	3.85 ± 0.87	−1.04	−1.46 to −0.63	<0.001	LM
Superior sagittal canal width	3.73 ± 1.51	3.49 ± 1.58	0.95	0.80 to 1.12	0.522	GLM
Middle sagittal canal width	3.42 ± 1.15	2.74 ± 1.01	0.79	0.69 to 0.91	0.001	GLM
Inferior sagittal canal width	4.72 ± 1.17	3.80 ± 0.85	0.80	0.73 to 0.87	<0.001	GLM
Length of the canal	9.63 ± 3.54	11.45 ± 2.88	1.66	0.42 to 2.90	0.009	LM
Nasopalatine canal angle	120.39 ± 10.57	113.82 ± 7.43	−6.47	−9.93 to −3.00	<0.001	LM

GLM, generalized linear models, estimate: adjusted ratio of means; LM, linear models, estimate: adjusted mean difference.

### 3.4 Morphometric characteristics of the bone anterior to the nasopalatine canal

With respect to the bone anterior to the nasopalatine canal, the length of the buccal crestal bone was significantly greater in the dentate group than in the completely edentulous group, with an adjusted mean difference of 4.66 mm (95% CI: 3.23–6.09; *p* < 0.001).

Similarly, the bone width measured 1 mm apical to the alveolar crest was significantly greater in the dentate group, with an adjusted ratio of means of 1.65 (95% CI: 1.35–2.02; *p* < 0.001), indicating an approximately 65% greater bone width than that in the completely edentulous group. The bone width measured 6 mm apical to the alveolar crest was also significantly greater in the dentate group (mean ratio = 1.24, 95% CI: 1.10–1.39; *p* < 0.001), corresponding to an approximately 24% increase relative to the completely edentulous group. The detailed results of the morphometric characteristics of the bone anterior to the nasopalatine canal are presented in Table 2.

**Table 2:** Morphometric characteristics of the bone anterior to the nasopalatine canal.

Variable	Completely edentulous group	Dentate group	Estimate	95% CI	*P* value	Model
Length of crestal buccal bone	13.91 ± 4.12	18.50 ± 3.12	4.66	3.23–6.09	<0.001	LM
The bone width measured 1 mm apical to the alveolar crest	2.12 ± 0.94	3.46 ± 2.13	1.65	1.35–2.02	<0.001	GLM
The bone width measured 6 mm apical to the alveolar crest	5.76 ± 2.03	7.06 ± 1.69	1.24	1.10–1.39	<0.001	GLM

LM, linear model; Estimate: adjusted mean difference; GLM, generalized linear model; estimate: adjusted ratio of means.

#### 3.4.1 Effect size interpretation

For the morphometric characteristics of the nasopalatine canal, canal length had a moderate effect (*d* = 0.52; partial *η*^2^ = 0.08), whereas canal angulation had a moderate-to-large effect (*d* = −0.73; partial *η*^2^ = 0.12). Among the sagittal canal dimensions, the superior sagittal width had a trivial effect (*d* = −0.13; partial *η*^2^ = 0.01), whereas the middle and inferior sagittal widths had moderate-to-large (*d* = −0.66; partial *η*^2^ = 0.09) and large effects (*d* = −0.92; partial *η*^2^ = 0.16), respectively. For the horizontal canal measurements, the superior horizontal width had a small effect (*d* = 0.24; partial *η*^2^ = 0.01), whereas the middle and inferior horizontal widths had moderate (*d* = −0.55; partial *η*^2^ = 0.07) and large effects (*d* = −0.98; partial *η*^2^ = 0.18).

With respect to the bone anterior to the nasopalatine canal, the length of the buccal crestal bone had the greatest effect among all the variables (*d* = 1.27; partial *η*^2^ = 0.29). The bone widths measured 1 mm and 6 mm apical to the alveolar crest also demonstrated large effects (*d* = 0.88; partial *η*^2^ = 0.15 and *d* = 0.80; partial *η*^2^ = 0.13, respectively).

Overall, the greatest anatomical differences between dentate and completely edentulous individuals were observed in terms of the buccal crestal bone length and inferior canal dimensions, whereas superior canal measurements.

Post hoc power analyses demonstrated adequate statistical power for the principal morphometric outcomes, whereas variables with trivial or small effect sizes exhibited correspondingly lower statistical power, consistent with the minimal between-group differences observed (Supplementary Table S1).

### 3.5 Morphological characteristics of the nasopalatine canal

#### 3.5.1 Coronal plane morphology

On the coronal plane, the single canal configuration and the Y-shaped configuration were the two most frequently observed morphologies in both groups. In the dentate group, the Y-shaped configuration was the most prevalent pattern, accounting for 51.9% of the cases. In contrast, the completely edentulous group predominantly presented a single canal configuration (64.8%). The parallel double canal configuration was rarely observed and was identified only in the completely edentulous group (5.6%).

The Y-shaped configuration was significantly more common in the dentate group than in the completely edentulous group (OR = 2.53; 95% CI: 1.08–6.11), indicating that dentate individuals were approximately 2.5 times more likely to exhibit this configuration. In contrast, the single canal configuration was not significantly different between the groups (*p* = 0.120). The parallel double-canal configuration, although detected only in the completely edentulous group, did not significantly differ (*p* = 0.243). Overall, these findings suggest that the greater prevalence of the Y-shaped configuration in dentate individuals contributed the most to the observed morphological differences between the two groups on the coronal plane.

#### 3.5.2 Sagittal plane morphology

On the sagittal plane, the cylindrical configuration was the most common nasopalatine canal morphology observed in the overall sample. In the dentate group, the cylindrical shape accounted for 61.1% of the cases, followed by the funnel-shaped configuration (20.4%), the banana-shaped configuration (11.1%), and the hourglass-shaped configuration (7.4%). In contrast, the completely edentulous group demonstrated a more even distribution of canal morphologies, with relatively higher proportions of banana-shaped canals (24.1%) and hourglass-shaped canals (22.2%), although the cylindrical configuration remained the most frequent morphology (29.6%).

Among the evaluated configurations, only the cylindrical shape demonstrated a statistically significant difference between the groups. Dentate individuals were approximately 3.7 times more likely to exhibit a cylindrical nasopalatine canal than completely edentulous patients were (OR = 3.68; 95% CI: 1.56–9.00). This association remained statistically significant after Bonferroni correction for multiple comparisons (adjusted *p* = 0.007). Conversely, the banana-shaped, hourglass-shaped, and funnel-shaped configurations did not significantly differ between the two groups. Among these, the hourglass-shaped configuration tended to have a lower prevalence in the dentate group (OR = 0.28; 95% CI: 0.06–1.02; *p* = 0.055), although this difference did not reach statistical significance after correction for multiple testing.

Table 3 presents the distributions of the morphological configurations of the nasopalatine canal.

**Table 3:** Morphological characteristics of the nasopalatine canal.

The canal configuration	Completely edentulous group (%)	Dentate group (%)	*P* value
On the coronal plane			0.019*
Single and broad cannal	35 (64.8%)	26 (48.1%)	
Y-shaped canals	16 (29.6%)	28 (51.9%)	
Two parallel canals	3 (5.6%)	0 (0.0%)	
On the sagittal plane			0.005*
Banana-shaped	13 (24.1%)	6 (11.1%)	
Hourglass	12 (22.2%)	4 (7.4%)	
Cylindrical	16 (29.6%)	33 (61.1%)	
Funnel	13 (24.1%)	11 (20.4%)	

* Fisher's exact test.

## 4 Discussion

With the advancement of CBCT, detailed three-dimensional evaluation of craniofacial anatomical structures has become possible, particularly for the nasopalatine canal, which exhibits considerable morphological variability and represents an important anatomical structure to consider during dental procedures in the anterior maxilla (1, 14). The present study was conducted to investigate potential differences in the anatomical characteristics of the nasopalatine canal and the bone region anterior to the canal between completely edentulous and dentate maxillae. To the best of our knowledge, this study represents the first investigation evaluating these anatomical structures in a Southeast Asian population while directly comparing dentate individuals with completely edentulous patients.

In the present study, the results indicated that the nasopalatine canal width tended to increase in completely edentulous patients, both in the buccolingual and mesiodistal dimensions. Notably, in this study, the buccolingual width was measured using the Frankfort horizontal plane as the reference plane. This measurement approach may result in slightly smaller canal diameter values than the actual anatomical diameter, which may partially explain the differences observed when the present findings are compared with those reported in previous studies (12, 15). Nevertheless, previous studies comparing partially edentulous and dentate individuals have reported that post-extraction alveolar bone resorption may affect the morphology and dimensions of the nasopalatine canal (12, 16). Conversely, some investigations have suggested that the diameter of the nasopalatine canal may remain relatively stable in edentulous individuals (7, 17). Such discrepancies across studies may be related to differences in measurement protocols, reference planes, imaging parameters, or population characteristics.

With respect to the length of the nasopalatine canal, the present study revealed mean values of 11.45 ± 2.88 mm in the dentate group and 9.63 ± 3.54 mm in the completely edentulous group, indicating a tendency toward reduced canal length in completely edentulous patients. This finding is consistent with the observations reported by Tözüm et al. (7), who similarly noted a decrease in canal height following tooth loss. With respect to nasopalatine canal angulation, our findings contrast with those of Keşkek et al., who reported decreased canal angulation in edentulous patients (18). This inconsistency may be explained by differences in angulation measurement protocols between the studies. Despite these methodological variations, both studies suggest that the nasopalatine canal tends to incline more anteriorly and inferiorly following tooth loss. The variability in reported values may be explained in part by differences in measurement protocols and landmark identification. In the present study, canal length was determined via reference points primarily derived from width-related anatomical landmarks, which may influence the final measurement. Consequently, the measured canal length may slightly underestimate the true anatomical length in certain morphological configurations. This effect is particularly evident in banana-shaped canals, where the curved trajectory of the canal may lead to smaller linear measurements when a straight reference axis is used. In contrast, for other canal morphologies, the discrepancy between the measured and actual canal lengths is expected to be minimal.

With respect to the nasopalatine canal morphology observed on the coronal plane, the single canal and Y-shaped configurations were the two most frequently encountered patterns in both study groups. In the dentate group, the Y-shaped configuration was the most prevalent morphology, accounting for 51.9% of the cases, which is consistent with the findings reported by Fukuda et al. (16). In contrast, the edentulous group demonstrated a predominance of the single canal configuration (64.8%), a distribution similar to that described by Özarslantürk et al. (11) in partially edentulous patients. The observed differences in canal morphology between dentate and completely edentulous individuals on the coronal plane may be related to post-extraction anatomical remodeling. Following tooth loss, alveolar bone resorption and expansion of the nasopalatine canal may occur, potentially altering the internal branching pattern of the canal. These structural changes may contribute to the increased prevalence of simplified canal configurations, such as the single canal type, in edentulous patients.

The classification of nasopalatine canal morphology on the sagittal plane in the present study followed a four-type system—cylindrical, funnel-shaped, hourglass-shaped, and banana-shaped—similar to the classification proposed by Fukuda et al. (16). Overall, the cylindrical configuration was the most frequently observed morphology in the entire sample, which is consistent with the findings reported in several previous CBCT studies (7, 12, 17). In the dentate group, the cylindrical type accounted for 61.1% of the cases, followed by the funnel-shaped configuration (20.4%), the banana-shaped configuration (11.1%), and the hourglass-shaped configuration (7.4%). In contrast, the completely edentulous group demonstrated a more evenly distributed pattern of canal morphologies, with relatively higher proportions of banana-shaped (24.1%) and hourglass-shaped (22.2%) canals, although the cylindrical type remained the most common configuration (29.6%). However, the distribution observed in the present study differs from that reported by Fukuda et al. (16), who reported the funnel-shaped canal to be the most prevalent morphology in maxillary specimens, followed by the cylindrical type. Similar findings were also reported by Mardinger et al. (19). These discrepancies may suggest that factors such as ethnic background, environmental influences, and dental status may significantly affect the morphological characteristics of the nasopalatine canal.

With respect to the bone region anterior to the nasopalatine canal, the present study demonstrated that the dentate group presented significantly greater buccal bone dimensions than did the completely edentulous group did, particularly in terms of bone width measured at 1 mm and 6 mm apical to the alveolar crest. A similar trend was reported by Tözüm et al. (7), although the measurement protocol differed. In their study, the width of the buccal bone was evaluated at three reference levels, namely, at the horizontal level of the crestal, middle, and the most apical point of the canal. Despite differences in measurement landmarks and methodological approaches, both studies consistently demonstrated that the buccal bone plate is thicker in dentate individuals than in edentulous patients. The values reported in the present study differ considerably from those described by Tözüm et al. (7), who reported mean bone heights of 19.97 ± 3.30 mm in dentate individuals and 16.38 ± 3.68 mm in edentulous patients. Nevertheless, both studies consistently reported that the buccal bone height anterior to the nasopalatine canal is greater in dentate individuals than in edentulous individuals, suggesting that tooth loss is associated with significant vertical and horizontal bone resorption in the anterior maxilla.

The findings of the present study indicate that completely edentulous patients tend to exhibit increased nasopalatine canal width, greater canal angulation, and a reduction in the surrounding anterior maxillary bone dimension. In addition, the results demonstrated an increase in the nasopalatine canal angle and a decrease in the buccal bone height anterior to the canal in completely edentulous individuals. These anatomical alterations may be largely attributed to alveolar bone resorption associated with aging and, more prominently, following tooth extraction (20). Recent evidence indicates that although the buccal bone anterior to the nasopalatine canal undergoes age-related resorption, the dimensions of the nasopalatine canal itself remain relatively stable with increasing age (21, 22). Therefore, the differences in canal dimensions observed among studies are likely multifactorial, reflecting the combined effects of tooth loss, age-related alveolar bone remodeling, and variations in the quantity and quality of the surrounding bone rather than aging alone. Furthermore, future investigations should incorporate potential confounding factors associated with alveolar bone resorption, including systemic conditions such as diabetes mellitus and osteoporosis, as well as patient-related oral factors, including the duration and etiology of tooth loss, previous prosthetic rehabilitation, and other oral health-related habits or conditions. Accounting for these variables may provide a more comprehensive understanding of the factors influencing the morphology and morphometric characteristics of the nasopalatine canal (22). Furthermore, the observed variations may also be influenced by several factors, including demographic characteristics, ethnic background, environmental influences. In the present study, the investigated population consisted of completely edentulous patients, whereas most previously published studies have primarily examined dentate or partially edentulous populations. Studies specifically focusing on completely edentulous individuals remain relatively limited worldwide. In addition, differences in measurement protocols, reference landmarks, and methods used to determine canal orientation and angulation may contribute to the variability in findings reported across different studies.

In recent years, implant placement involving the nasopalatine canal region has become increasingly utilized in full-arch rehabilitation for patients with severe maxillary bone resorption. This anatomical site has been proposed as a feasible implant anchorage area, with studies suggesting that it does not pose a substantial surgical risk and may contribute to improved load distribution in full-arch prosthetic restorations (5, 6). Currently, two principal surgical approaches have been described for implant placement in the nasopalatine canal region. The first technique involves removal of the nasopalatine neurovascular bundle followed by direct implant placement within the canal. In this approach, the implant engages the bony walls of the nasopalatine canal; therefore, the implant diameter is largely determined by the canal dimensions (23). In the present study, the largest mean canal diameter measured 4.72 ± 1.17 mm in the buccolingual direction and 4.83 ± 1.24 mm in the mesiodistal direction, which falls within the commonly available range of implant diameters of approximately 3–6 mm. The second technique involves implant placement in the bone anterior to the nasopalatine canal while preserving the neurovascular bundle within the canal (24). In this approach, successful implant placement depends largely on the available bone volume and density in the anterior bone region. Consequently, careful selection of the implant dimension is needed to ensure adequate primary stability while avoiding injury to the neurovascular structures of the nasopalatine. Moreover, the observed anatomical variations highlight the necessity of evaluating both the vertical and horizontal dimensions of the nasopalatine canal region during preoperative CBCT assessment. Such a comprehensive three-dimensional evaluation should be regarded as a prerequisite for determining the initial implant dimensions and developing a patient-specific implant treatment plan. A recent systematic review by De Mello et al. (4) reported implant survival rates ranging from 84.6% to 100% for implants placed in the nasopalatine canal region. Among the included studies, transient neurosensory disturbances were reported in three studies, whereas permanent sensory impairment was documented in only one study. Although implant placement in this region appears to be a predictable treatment option with high success rates, the potential for neurovascular complications should not be overlooked. Therefore, careful assessment of the morphological variability of the nasopalatine canal during preoperative CBCT evaluation is essential to minimize the risk of neurovascular injury during implant surgery. Similar to the routine assessment of bifid and trifid mandibular canals, identifying anatomical variations of the nasopalatine canal before surgery may facilitate individualized treatment planning and contribute to safer, more predictable surgical outcomes (25).

Our findings demonstrated that completely edentulous patients exhibited significantly greater nasopalatine canal widths, with a mean difference of approximately 1 mm compared with dentate individuals. From a clinical perspective, this difference may have important implications for implant placement within the nasopalatine canal, as a wider canal could necessitate the use of implants with a diameter at least 1 mm greater to achieve comparable bone engagement. Consequently, certain clinical situations may require the use of wide-diameter implants (>5 mm) rather than the medium-diameter implants (approximately 4 mm) reported in previous studies of implants placed within the nasopalatine canal, such as that by Raghoebar et al. (26). In addition, the significantly shorter canal length observed in completely edentulous patients (mean difference, 1.66 mm) may influence the selection of implant length. Specifically, shorter implants (e.g., 10 mm instead of 11.5–12 mm) may be more appropriate in selected cases to accommodate the reduced canal length while maintaining adequate anatomical safety margins during implant placement. Nevertheless, these considerations should be interpreted with caution, as the present study was based on anatomical CBCT measurements rather than clinical implant outcomes.

Several limitations of this study should be acknowledged. First, comprehensive clinical information was not available for all patients in the dataset, including oral health status, systemic conditions (e.g., diabetes mellitus and osteoporosis), the duration and etiology of tooth loss, and previous prosthetic rehabilitation. The absence of these data precluded adjustment for potentially important confounding factors that may influence alveolar bone remodeling and the morphology of the nasopalatine canal. These factors may play a role in the extent of alveolar bone remodeling and nasopalatine canal morphology and therefore could have influenced the results. Second, the study was conducted with a relatively limited sample size, and the data were obtained from a single center, which may limit the representativeness of the study population and reduce the generalizability of the findings. Future studies with larger multicenter samples, balanced demographic characteristics, and comprehensive clinical data are needed to further validate and expand upon the findings of the present study. Furthermore, the use of a single standardized CBCT imaging protocol precluded the evaluation of whether variations in imaging parameters, including voxel size, field of view (FOV), and slice thickness, affect the accuracy of morphometric measurements. Future studies incorporating multiple imaging protocols are warranted to validate the reproducibility and generalizability of these findings.

## 5 Conclusion

The present CBCT-based study demonstrated that tooth loss is associated with significant anatomical alterations in the nasopalatine canal and the surrounding anterior maxillary bone. Specifically, completely edentulous patients exhibited increased canal width and angulation, whereas the canal length and the anterior bone dimension to the canal were reduced compared with those of dentate individuals. In addition, the nasopalatine canal showed substantial morphological variability. In the coronal plane, the Y-shaped configuration predominated in dentate individuals, whereas the single canal configuration was more common in completely edentulous patients. On the sagittal plane, the cylindrical morphology represented the most frequent configuration across the entire sample.

These findings underscore the dynamic anatomical changes occurring in the anterior maxilla following tooth loss and highlight the importance of comprehensive CBCT evaluation for individualized planning of implant treatment in this region. Given the considerable interindividual variability in canal morphology and surrounding bone dimensions, patient-specific anatomical assessment should be considered essential for optimizing implant placement and minimizing surgical complications in the anterior maxilla.

## Data Availability

The raw data supporting the conclusions of this article will be made available by the authors, without undue reservation.
