# Supplementary material for: Morphological and morphometric characteristics of the nasopalatine canal in dentate and completely edentulous patients: a cone-beam computed tomography cross-sectional study
Source: Front Oral Health. 2026 Jul 22;7:1899731. doi: 10.3389/froh.2026.1899731 (PMC13437972; doi:10.3389/froh.2026.1899731)
Supplement: Supplementary file 1 [file Table1.docx]

**Supplementary Table S1. Post hoc power analysis for morphometric outcomes comparing dentate and completely edentulous participants**

| Variable | Cohen's d | Partial η² | Achieved power |
| --- | --- | --- | --- |
| Canal length | 0.52 | 0.08 | 0.763 |
| Canal angulation | 0.73 | 0.12 | 0.964 |
| Superior sagittal width | 0.13 | 0.01 | 0.103 |
| Middle sagittal width | 0.66 | 0.09 | 0.925 |
| Inferior sagittal width | 0.92 | 0.16 | 0.997 |
| Superior horizontal width | 0.24 | 0.01 | 0.235 |
| Middle horizontal width | 0.55 | 0.07 | 0.808 |
| Inferior horizontal width | 0.98 | 0.18 | 0.999 |
| Buccal crestal bone length | 1.27 | 0.29 | 1.000 |
| Buccal bone width (1 mm) | 0.88 | 0.15 | 0.995 |
| Buccal bone width (6 mm) | 0.80 | 0.13 | 0.985 |

*Post hoc power was calculated using the observed Cohen's d values, the final sample size (54 participants per group), and a two-sided α level of 0.05.*
